# The use of HaloTag-based technology in flow and laser scanning cytometry analysis of live and fixed cells

**DOI:** 10.1186/1756-0500-4-340

**Published:** 2011-09-09

**Authors:** Elena I Kovalenko, Shahin Ranjbar, Luke D Jasenosky, Anne E Goldfeld, Ivan A Vorobjev, Natasha S Barteneva

**Affiliations:** 1 Immune Disease Institute and Program in Cellular and Molecular Medicine, Children's Hospital Boston and Harvard Medical School, Boston, MA, USA; 2 Shemyakin-Ovchinnikov Institute of Bioorganic Chemistry, Russian Academy of Sciences, Moscow, Russia; 3 National Hematological Scientific Center, Moscow, Russia; 4 Belozersky Institute of Physico-Chemical Biology, Moscow State University, Moscow, Russia; 5 Department of Pediatrics, Harvard Medical School, Boston, MA, USA

## Abstract

**Background:**

Combining the technologies of protein tag labeling and optical microscopy allows sensitive analysis of protein function in cells.

**Findings:**

Here, we describe development of applications using protein tag technology (HaloTag (HT)-based) for flow and laser scanning cytometry (LSC). Cell lines, expressing recombinant surface β1-integrin-HT and HT-p65 fusion protein, and a CD4 T cell line (Jurkat) infected with human immunodeficiency virus type 1 (HIV-1) reporter virus expressing the unfused HT (HIV-1_Lai-Halo_), were stained with different HT ligands and successfully detected by flow cytometers equipped with 488 and 561 nm lasers as well as a laser scanning cytometer (equipped with 488 and 405 nm lasers) alone or combined with cell cycle and viability markers.

**Conclusions:**

Use of HT technology for cytometric applications has advantages over its use in microscopy as it allows for the statistical measurement of protein expression levels in individual cells within a heterogeneous cell population in combination with cell cycle analysis. Another advantage is the ability of the HaloTag to withstand long fixation and high concentration of fixative, which can be useful in research of infectious agents like HIV and/or mycobacteria.

## Findings

One limitation of the fluorescent proteins commonly used in the generation of fusion proteins is that new constructs must be created if different colors are required for analysis. In addition to the labor involved, changing the fusion partner can result in dysregulated localization and/or affect the activity of the protein being analyzed [1]. Another problem is that fluorescent proteins are often prone to fixation, making difficult to combine flow cytometric analysis with cell cycle studies or research of infection agents like HIV or mycobacteria which requiring high percentage of fixation and long fixative time. An alternative approach is to use a fusion partner like HaloTag, SNAP-Tag, FIAsH, or others [2-5] that can be "tagged" later with an exogenous fluorescent ligand. Labeling of these protein tags with small synthetic ligands depends on the formation of stable complexes between biarsenical compounds and peptides containing a tetracysteine thiol motif [6], and is adaptable to a wide range of cell types [7]. The "tag" technologies have been utilized in diverse experimental procedures, but mainly for *in vitro *and *in vivo *imaging of cells with microscopy and immunocytochemistry [4,7,8].

Here we describe the successful application of flow cytometry and laser scanning cytometry (LSC) to cells expressing HT constructs. The HT protein is an engineered monomeric haloalcane dehalogenase from *Rhodococcus rhodochrous *capable of covalent binding to ligands of interest. The HT ligands harbor reactive linkers that covalently bind to the HT protein and fluorescent reporter groups or affinity handles such as biotin, Oregon Green, tetramethylrhodamine (TMR) and others.

We illustrate the utility of this approach with several different systems: i) surface staining of a U2OS human osteosarcoma cell line that is stably transfected with a vector encoding β1-integrin-HaloTag7 (U2OS-β1Int-HT7); ii) intracellular staining of Jurkat CD4 T cells infected with an HIV-1 reporter virus (HIV-1_Lai-Halo_) that encodes an unfused HT; and iii) intracellular staining of a HEK-293 cell line that expresses a HT fusion with the nuclear factor (NFκB) p65 subunit. The β1Int-HT7 construct is well expressed and tolerated in multiple mammalian cell types [9].

**Figure 1:**
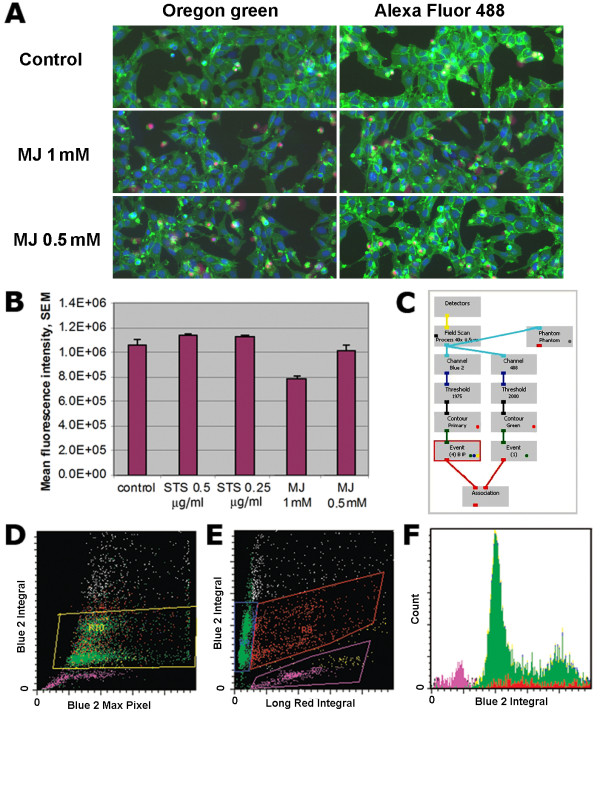
**Laser scanning cytometry analysis of β1-integrin expression and cell cycle staining in the U2OS-β1Int-HT7 cell line**. **A**. Field images for U2OS-β1Int-HT7 cells treated with MJ (1 mM and 0.5 mM) or left untreated for 16 h and then stained with Oregon Green and Alexa 488 HT ligands. Blue is cell nuclei stained with Hoechst 33342; green is the β1 integrin-HT fusion protein stained with Oregon Green HT ligand; red is nuclei of necrotic cells stained with PI. Images were acquired with two lasers (405 and 488 nm). Two types of primary contouring were applied to the samples: blue object image (based on nucleus contouring) and phantom. The same pattern in fluorescence intensity levels was achieved with Oregon Green and Alexa 488 HT ligands. **B**. Analysis of green cell fluorescence based on phantom primary contouring in samples treated with different doses of STS (0.25 μg or 0.5 μg/ml) or MJ (0.5 or 1 mM), or left untreated. **C**. The protocol of image acquisition on the laser scanning cytometer. **D, E, F**. Cell cycle analysis was performed by Hoechst staining. Dot-blots are shown of cell populations gated on the base of blue (D) and red (E) fluorescence. A cell cycle histogram and pre-G1-peak (apoptotic subpopulation) defined on the basis of Hoechst staining (F).

We first took advantage of the statistical possibilities associated with laser scanning cytometry of adherent cells. U2OS-β1Int-HT7 cells were seeded at a density of 0.5x10^6 ^cells/ml in a 96-well flat-bottom plate in a volume of 100 µl/well and were grown overnight in DMEM supplemented with 10% fetal bovine serum (FBS), 2 mM *L*-glutamine, 10 mM HEPES, 100 U/ml penicillin, 100 µg/ml streptomycin (all from Gibco products, Invitrogen, Carlsbad, CA), in a humid atmosphere of 5% CO_2 _at 37°C. Cells were treated with methyl jasmonate (MJ) (1 or 0.5 mM), staurosporine (STS) (0.5 or 0.25 µg/ml) (both Sigma, St-Louis, MO), or left untreated for 16 h, and then washed with warm medium. The cells were then stained with Oregon Green (0.5 μM) or Alexa 488 (1.0 μM) HT ligands (both - Promega, Madison, WI) for 30 min and carefully washed with warm medium; Hoechst 33342 (5 µg/ml), and propidium iodide (PI) (1 µg/ml) were added for 30 min before analysis. Fluorescence was excited with the 488 and 405 nm lasers. The intensities of maximal pixel and integrated fluorescence were quantified and recorded for each cell. At least 3,000 cells were measured per well. Surface β1-integrin expression was easily detected using both Oregon Green and Alexa 488 HT ligand staining (Figure 1A). Cellular green (HT ligand Oregon Green), far red (PI), and blue (Hoechst) fluorescence emissions were measured simultaneously in the same cells in situ utilizing standard filter settings and an iCys cytometer (Compucyte, Westwood, MA) (Figure 1C). Mean fluorescence intensity (MFI) of β1-integrin expression was calculated per thousand cells in the 96-well plate in a high-throughput manner (Figure 1B) and correlated with the level of apoptosis (pre-G1-peak). β1-integrin expression was reduced in a concentration-dependent manner upon addition of MJ (1 and 0.5 mM), but not STS (0.25 and 0.5 μg) (Figure 1B). U2OS cells transfected with β1-integrin appeared to be much more resistant to apoptosis induced by STS (0.5 µg/ml) (data not shown). LSC analysis of U2OS-β1Int-HT7 cells enabled pre-G1 peak quantitation, cell viability, and fluorescent intensity of β1-integrin expression to be measured simultaneously (Figure 1 D, E, and 1F).

**Figure 2:**
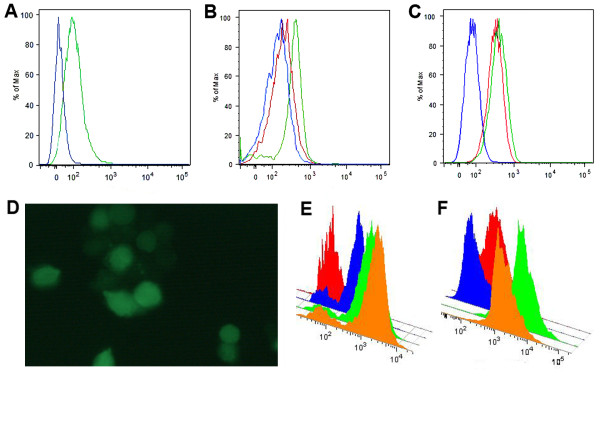
**HT ligand-based FACS analysis of HIV-1**_**Lai-Halo**_**-infected cells and HEK-293 cells expressing a HT-p65 fusion protein**. **A, B, C **FACS analysis of Jurkat cells infected with HIV-1_Lai-Halo _with a FACSAria cytometer equipped with a 488- and 561-nm lasers._. _**A**. Oregon Green ligand staining. (blue: uninfected cells; green: virus-infected cells); **B**. TMR ligand staining (blue: uninfected, unstained cells; red: uninfected, stained cells; green: infected, stained cells). **C**. FACS analysis of fixed Jurkat cells stained with TMR ligand comparing with unfixed cells (blue: uninfected cells; red: virus-infected cells fixed with PFA; green: virus-infected, unfixed cells). Data were acquired with DiVa 6.1 software 5 days postinfection and analyzed off-line with the FlowJo program (Treestar, Ashland, OR). **D**. Microscopic analysis of Jurkat cells infected with HIV-1_Lai-Halo _and stained with the Oregon Green HT ligand. **E**. FACS analysis of HEK-293 cells expressing the HT-p65 fusion protein and stained with different doses of Alexa 488 HT ligand (red: unstained cells; blue, green and orange: 0.25, 0.5, and 1.0 μM, respectively). **F**. Control HEK-293 cells unstained and stained with Oregon Green HT-ligand (red and blue, respectively) and cells transfected with p65-expressing plasmid and stained with 0.5 and 1.0 μM Oregon Green HT-ligand (green and orange, respectively).

We next tested the utility of HT system for intracellular flow cytometry. An HIV-1 infectious molecular clone encoding the HT was constructed by introducing the HT protein (unfused to any other protein sequence) derived from the plasmid pHT2 (Promega, Madison, WI, USA) into the HIV-1_Lai _(NIH AIDS Research & Reference Reagent Program) background. This new infectious molecular clone was designated HIV-1_Lai-Halo _and was constructed in a manner similar to an EGFP reporter virus described previously [10]. Jurkat T cells were cultured in RPMI 1640 medium with 2 mM L-glutamine (BioWhittaker, Inc., Walkersville, MD) supplemented with 10% heat inactivated FBS. Cells (0.5 × 10^5 ^cells/ml) were infected with HIV-1_Lai-Halo _(1000 TCID_50_) and incubated at 37°C and 5% CO_2 _for 5 days, after which the cultures were terminated. Infected and uninfected (control) cells were labeled with HaloTag TMR (5 μM) or HaloTag Oregon Green (1 μM) ligand in medium for 40 min at 37°C and then washed three times with phosphate buffered saline (PBS). Cells were further incubated with fresh medium for 40 min at 37°C before they were washed with PBS (three times) and fixed with 4% paraformaldehyde (PFA) plus 0.2 M sucrose in PBS for 2 h before analysis. Cells were analyzed using a FACSAria cytometer (BD Biosciences, San Jose CA) equipped with 488 nm and/or 561 nm lasers. Virus-derived HT protein was easily detected after staining with Oregon Green (Figure 2A and 2D) or TMR (Figure 2B). Fixation of cells with 4% PFA for 2 h had little effect on fluorescence intensity of the infected cells stained with TMR HT ligand (Figure 2C) and no effect on Oregon Green HT ligand (data not shown).

We also performed intracellular staining of the HEK-293 cell line stably expressing HT-p65 fusion protein. Cells were fixed with 1% PFA for 1 h at 4°C following a 30-min permeabilization at 4°C with 70% ethanol and staining with the Alexa 488 HT ligand (30 min × 37°C w/o fixation for Oregon Green). As shown in Figures 2E and 2F, ligand concentration can be optimized in order to achieve a good signal-noise ratio.

Analysis of HT expression using cytometers equipped with green-yellow lasers (laser excitation 561 nm) and green lasers (534 nm) enables the use of a versatile set of ligands, including TMR (494_Ex_/516_Em_) or HT-DiAcFAM ligand (494_Ex_/526_Em_). Before introduction of green-yellow and green lasers in the standard cytometer optical configuration, the availability of HT ligands for cytometry applications was limited by HT ligands excited with the 488 nm laser (Oregon Green and Alexa 488) and biotin-conjugated ligands. However, because biotin is an essential co-factor in the cytosol and in mitochondria, the use of biotin-conjugated ligands may result in a significant level of nonspecific binding, especially in intracellular applications.

The use of interchangeable ligands that recognize the HT and emit in green and red ranges makes it possible to modify a particular experiment in order to (1) avoid undesirable spectral overlapping; (2) decrease autofluorescence with TMR or other red-emitting HT ligands; (3) manipulate colors interchangeably for surface or intracellular staining without the need to generate additional plasmids encoding new fusion partner combinations; and (4) perform extensive cell fixation if infectious agents that require inactivation like HIV or *Mycobacterium tuberculosis *are being studied. The cytometric approach described herein should be applicable to other "tagged" proteins that utilize a diverse selection of fluorescent ligands (SNAP-tag, etc.) and to a wide range of host cells. In addition, since the HT ligand is connected by a stable covalent bond to its target protein, the fluorescently labeled HT fusion protein can be characterized by SDS-PAGE without loss of fluorescent signal (4).

The combination of HT technology with flow and imaging cytometry analysis has major advantages over microscopy-based methods. As shown here, these cytometric methods allow investigators to statistically assess the expression level of multiple proteins in individual cells within a heterogeneous cell population, and can be combined with cell cycle analysis, viability evaluation, and cell surface/intracellular staining, although nonspecific binding of HT ligands to untagged cellular components remains an issue. Taken together, the newly implemented applications for HT technology described herein should promote the adoption of rapid, efficient and quantitative multiplexing of "tagged" proteins with cell cycle dyes and fluorochrome-conjugated antibodies.

## List of abbreviations used

β1-integrin-HT7: vector encoding β1-integrin-HaloTag7; DiAcFAM: diacetyl derivative of fluorescein; EGFP: enhanced green fluorescent protein; FBS: fetal bovine serum; FIAsH: fluorescein arsenical hairpin; HIV: human immunodeficiency virus; HIV-1_Lai-Halo_: human immunodeficiency virus type 1 (HIV-1) reporter virus expressing the unfused HT; HT: Halotag; LSC - laser scanning cytometry; MFI: mean fluorescent intensity; MJ: methyl jasmonate; PBS: phosphate buffered saline; PFA: paraformaldehyde; PI: propidium iodide; SDS-PAGE: SDS-polyacrylamide gel electrophoresis; SNAP-tag: is a 20 kDa mutant of the human DNA repair protein O6-alkylguanine-DNA alkyltransferase (hAGT) that reacts specifically and rapidly with benzylguanine and benzylchlopyrimidine derivatives carrying a variety of different synthetic fluorophores; STS: staurosporine; TMR: tetramethylrhodamine.

## Competing interests

The authors declare that they have no competing interests.

## Authors' contributions

EK and SR designed the research, performed the experiments, analyzed the results and drafted the paper. LDJ created HIV-1_Lai-Halo _construct and contributed in data interpretation and report writing. AEG supervised SR and LDJ, coordinated the research, provided funding. IAV contributed in design of research, data interpretation and commented on manuscript. NSB designed and coordinated research, analyzed the data, wrote the manuscript and provided funding. All authors read and approved the final manuscript.

## References

[B1] Lisenbee CS Karnik SK Trelease RN Overexpression and mislocalisation of a tail-anchored GFP redefines the identity of peroxisomal ER Traffic 2003 4 491 501 10.1034/j.1600-0854.2003.00107.x 12795694

[B2] Keppler A Kindermann M Gendreizig S Pick H Vogel H Johnson K Labeling of fusion proteins of O6-alkylguanine-DNA alkyltransferase with small molecules in vivo and in vitro Methods 2004 32 437 444 10.1016/j.ymeth.2003.10.007 15003606

[B3] Tirat A Freuler F Stettler T Mayr LM Leder L Evaluation of two novel tag-based labeling technologies for site-specific modification of proteins Inter J Biolog Macromolecules 2006 39 66 76 10.1016/j.ijbiomac.2006.01.012 16503347

[B4] Los GV Wood K The HaloTag: a novel technology for cell imaging and protein analysis Methods Mol Biol 2007 356 195 208 16988404 10.1385/1-59745-217-3:195

[B5] Gautier A Juillerat A Heinis C Correa IR Kindermann M Beaufils F Johnsson K An engineered protein tag for multiprotein labeling in living cells Chem Biol 2008 15 128 136 10.1016/j.chembiol.2008.01.007 18291317

[B6] Griffin BA Adams SR Tsien RY Specific covalent labeling of recombinant protein molecules inside live cells Science 1998 281 269 272 9657724 10.1126/science.281.5374.269

[B7] Lang C Schulze J Mendel RR Hansch R HaloTag: a new versatile reporter gene system in plants J Experiment Botany 2006 57 2985 2992 10.1093/jxb/erl065 16873446

[B8] Schroeder J Benink H Dyba M Los GV In vivo labeling method using a genetic construct for nanoscale resolution microscopy Biophysical J 2008 96 L01 3 10.1016/j.bpj.2008.09.032 PMC2710050 19134467

[B9] Svendsen S McMillan E Zimprich C Swendsen CN Los GV HaloTag protein: a novel reporter protein for human neural stem cells Promega Notes 2007 95 20 22

[B10] Brown A Gartner S Kawano T Benoit N Cheng-Mayer C HLA-A2 down-regulation on primary human macrophages infected with an M-tropic EGFP-tagged HIV-1 reporter virus J Leukoc Biol 2005 78 675 685 10.1189/jlb.0505237 16000390

